# Therapeutic nanobodies against SARS-CoV-2 and other pathogenic human coronaviruses

**DOI:** 10.1186/s12951-024-02573-7

**Published:** 2024-05-31

**Authors:** Yang Yang, Fang Li, Lanying Du

**Affiliations:** 1 Roy J. Carver Department of Biochemistry, Biophysics and Molecular Biology, Iowa State University, Ames, IA USA; 2 Department of Pharmacology, University of Minnesota Medical School, Minneapolis, MN USA; 3 Center for Coronavirus Research, University of Minnesota, Minneapolis, MN USA; 4 Institute for Biomedical Sciences, Georgia State University, Atlanta, GA USA

**Keywords:** Pathogenic coronaviruses, SARS-CoV-2, SARS-CoV, MERS-CoV, Spike protein, Receptor-binding domain, Therapeutic antibodies

## Abstract

Nanobodies, single-domain antibodies derived from variable domain of camelid or shark heavy-chain antibodies, have unique properties with small size, strong binding affinity, easy construction in versatile formats, high neutralizing activity, protective efficacy, and manufactural capacity on a large-scale. Nanobodies have been arisen as an effective research tool for development of nanobiotechnologies with a variety of applications. Three highly pathogenic coronaviruses (CoVs), SARS-CoV-2, SARS-CoV, and MERS-CoV, have caused serious outbreaks or a global pandemic, and continue to post a threat to public health worldwide. The viral spike (S) protein and its cognate receptor-binding domain (RBD), which initiate viral entry and play a critical role in virus pathogenesis, are important therapeutic targets. This review describes pathogenic human CoVs, including viral structures and proteins, and S protein-mediated viral entry process. It also summarizes recent advances in development of nanobodies targeting these CoVs, focusing on those targeting the S protein and RBD. Finally, we discuss potential strategies to improve the efficacy of nanobodies against emerging SARS-CoV-2 variants and other CoVs with pandemic potential. It will provide important information for rational design and evaluation of therapeutic agents against emerging and reemerging pathogens.

**Graphical abstract:**

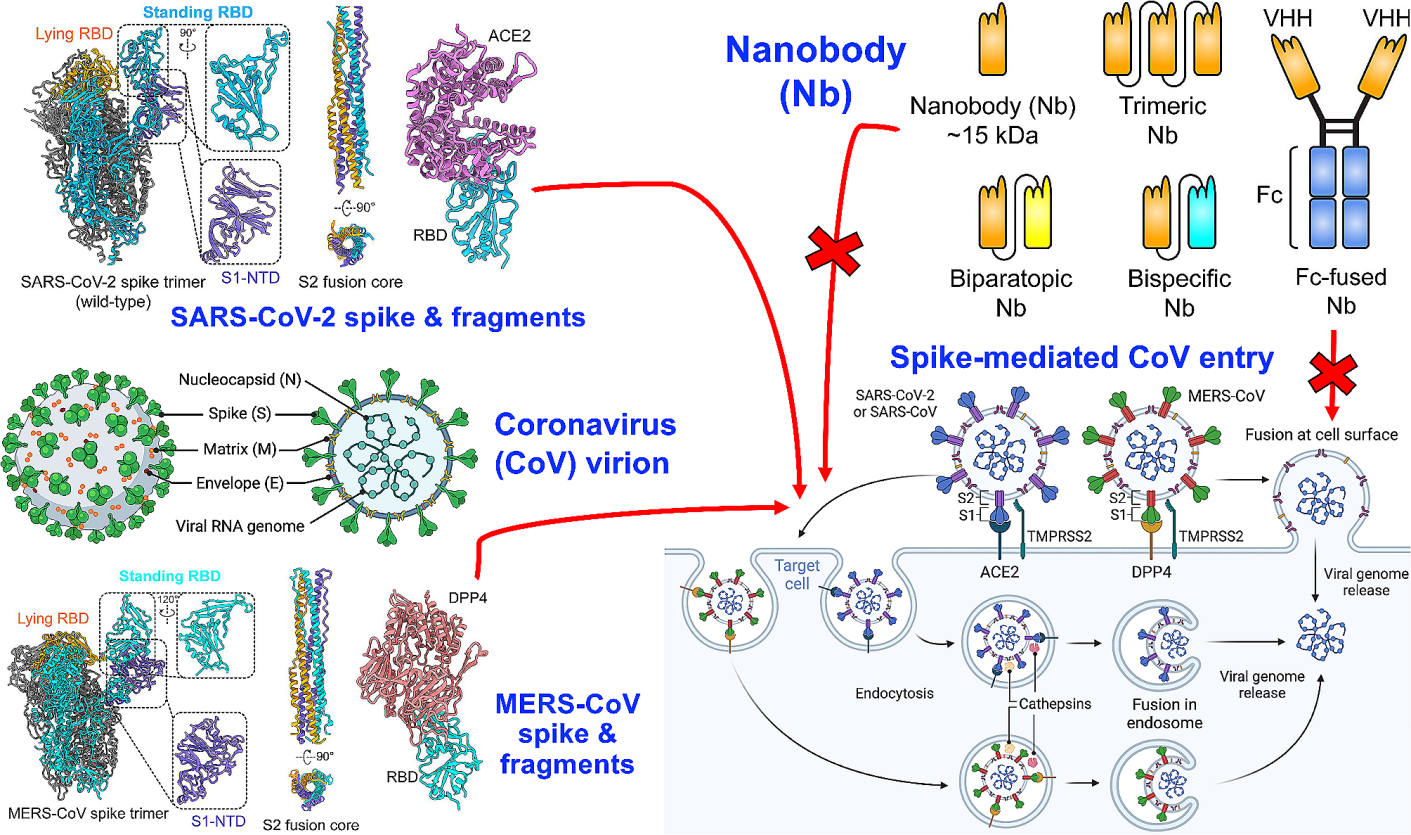

## Background

Coronaviruses (CoVs) are a large group of viruses belonging to the *Orthocoronavirinae* subfamily (family, *Coronaviridae*; order, *Nidovirales*), which cause diseases in humans and animals [1]. The *Orthocoronavirinae* subfamily comprises four genera: alpha-CoV, beta-CoV, gamma-CoV, and delta-CoV [1, 2]. Seven CoVs have been identified in humans, four of which (229E, NL63 (Alpha-CoV), OC43, and HKU1 (Beta-CoV)) cause mild respiratory illnesses, asymptomatic infections, or colds [3]. The other three CoVs belonging to the beta-CoV genera are severe acute respiratory symptom (SARS)-CoV, Middle East respiratory symptom (MERS)-CoV, and SARS-CoV-2; all are pathogenic and caused the SARS outbreak, the MERS outbreak, and the CoV Disease 2019 (COVID-19) pandemic in 2002–2003, 2012, and 2019, respectively [4–8]. SARS-CoV-2 and SARS-CoV are sarbecoviruses, whereas MERS-CoV is a merbecovirus [9, 10].

SARS (caused by SARS-CoV), which led to the first major emerging outbreak of the 21st century, was first reported in 2002 and emerged in February 2003, infecting 8,422 people and killing 916 (> 10% fatality) across 29 countries [7, 11]. SARS spreads mainly through human-to-human contact, mostly via respiratory droplets [11]. SARS-CoV is zoonotic, i.e., it originated from natural reservoirs such as bats, and transmited through intermediate hosts such as palm civets and raccoon dogs before infecting humans [12–14]. SARS infection causes a severe respiratory syndrome and pathological changes in lung tissues [15, 16]. Although have been no new SARS cases since 2004, some SARS-related CoVs from other species share the same receptor of SARS-CoV [17]; these viruses may have pandemic potential in the future. No vaccines and therapeutic agents have been approved for use against SARS-CoV; hence, more effort is needed to develop countermeasures to prevent reoccurrence of SARS and SARS-related illnesses.

MERS, caused by MERS-CoV, was first identified in Saudi Arabia in 2012, and has since been reported in at least 27 countries, causing 936 deaths among 2,605 infected cases (> 35% fatality) [6, 18]. MERS-CoV is a zoonotic CoV, which likely utilizes bats as its natural host and dromedary camels as the intermediate host [10]. Different from SARS-CoV and SARS-CoV-2, MERS-CoV has limited human-to-human transmissibility but can be transmitted through community or healthcare settings [19, 20]. MERS-CoV infects humans through close contact with infected camels or people [21, 22]. In addition, some MERS-related CoVs identified in bats share the same receptor as MERS-CoV [23], and thus have pandemic potential in the future. Currently, no vaccines or therapeutic agents have been authorized or approved for prevention and/or treatment of MERS-CoV infection. Again, continuous effort is needed to develop effective preventive and treatment strategies against MERS-CoV and MERS-related CoV infections.

SARS-CoV-2, the causative agent of COVID-19 pandemic, was first reported in December 2019 [4]. Unlike SARS-CoV and MERS-CoV, SARS-CoV-2 has superior human-to-human transmissibility; indeed, as of April 07, 2024, there have been > 7.044 million deaths among > 775 millions of infected cases (fatality rate, > 0.9%) [24]. Different from SARS-CoV and MERS-CoV, SARS-CoV-2 undergoes rapid and continuous mutation, resulting in at least five variants of concern (VOCs), i.e., Alpha, Beta, Gamma, Delta, and Omicron (Omicron is further divided into several important subvariants: BA.1, BA.2, BA.3, BA.4, BA.5, XBB.1.5, EG.5, BA.2.86, and HV.1, with the most recent dominant subvariants being JN.1 and KP.2) [25, 26]. Although a number of vaccines and therapeutic agents (antibodies and drugs) have been approved or authorized for prevention and/or treatment of SARS-CoV-2 infection in humans, their neutralizing or inhibitory activity against SARS-CoV-2 VOCs, particularly dominant subvariants, wanes very rapidly [27–32]. This is mainly due to numerous mutations within the S protein, particularly the receptor-binding domain (RBD), of these variants [33, 34]. As a result, effective universal vaccines and treatment strategies need to be developed to control current and future SARS-CoV-2 variants with pandemic potential.

### Structures and viral proteins of pathogenic human CoVs

**Fig. 1:**
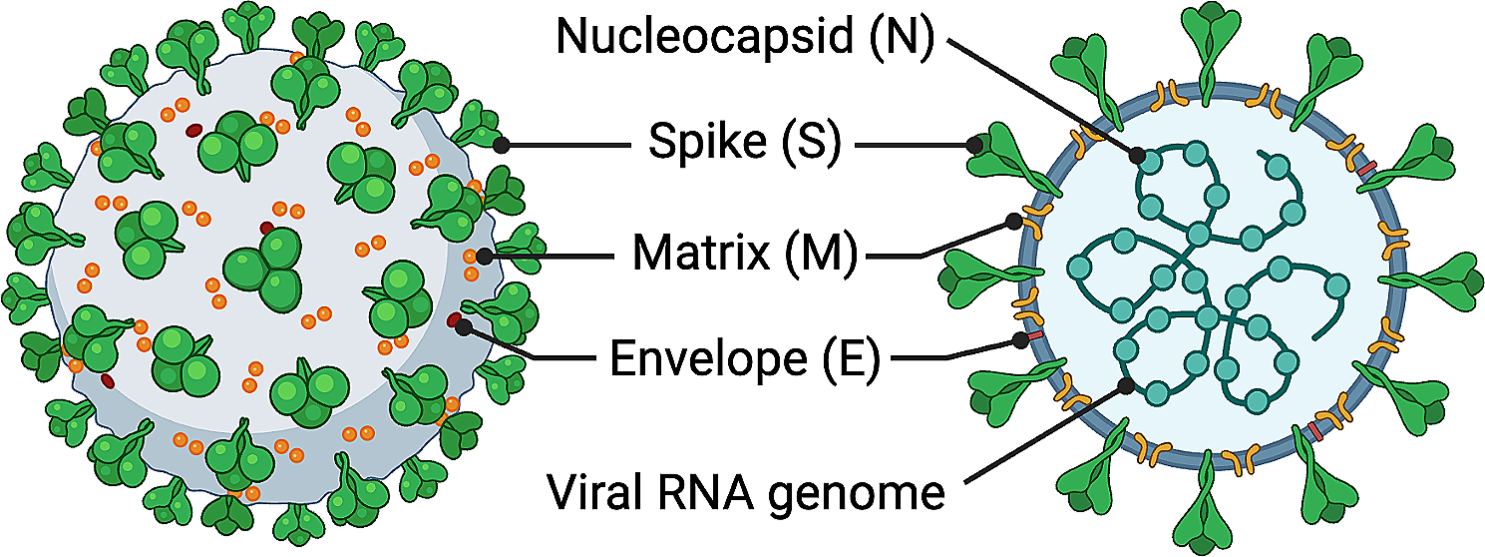
Schematic structure of coronavirus virion. The viral nucleocapsid (N), spike (S), matrix or membrane (M), envelop (E) proteins, and viral RNA genome are indicated

Similar to other CoVs, the pathogenic human CoVs (SARS-CoV, MERS-CoV, and SARS-CoV-2) are enveloped, single-stranded and positive-sense RNA viruses with a viral genome of > 25 kb in length (Fig. 1) [3, 7, 35]. The genome of these CoVs encodes a non-structural replicase polyprotein, which is translated from open-reading frames (ORFs) and further cleaved into various non-structural proteins (NSPs), including main protease (Mpro, NSP5) and papain-like protease (PLpro, NSP3) [3, 7]. The viral genome also encodes four major structural proteins, in the order of spike (S), envelope (E), membrane (M), and nucleocapsid (N) (Fig. 1), as well as a series of accessory proteins, which are named differently in various CoVs. The accessory proteins for SARS-CoV are 3a, 3b, 6, 7a, 7b, 8a, 8b, and 9b, those for MERS-CoV are 3, 4a, 4b, 5, and 8b, and those for SARS-CoV-2 are 3a, 6, 7a, 7b, 8, and 10 [3, 36].

The above viral proteins play different roles in the replication and life cycle of CoV. For example, the Mpro and PLpro are responsible for cleaving the viral polyprotein into the NSPs that play a role in regulating viral replication, spread, and innate immunity [37, 38]. The N protein is involved in binding genomic RNA and subsequent packaging of viruses into a ribonucleoprotein complex, thereby facilitating virion transcription and assembly [39]. Serving as an important membrane component, the M protein forms a dimer that mediates virus assembly, budding, and release [40]. The E protein serves as a pH-dependent cation channel, and is important for viral pathogenesis and assembly [41]. Together with the M protein, the E protein regulates maturation and retention of the S protein, thereby promoting assembly of SARS-CoV-2 viral particles [42]. Accessory proteins ORF3a and ORF7a downregulate surface expression of host major histocompatibility complex class I molecules via different mechanisms, whereas ORF8 is presented as a homodimer that plays a potential role in intracellular transport or extracellular signaling during viral infection [43, 44]. Consequently, several of these proteins are targets for development of anti-COVID-19 therapeutics or vaccines designed to block viral replication or infection processes [45–47].

### The S protein receptor-binding domain, and the receptors used by pathogenic human CoVs

**Fig. 2:**
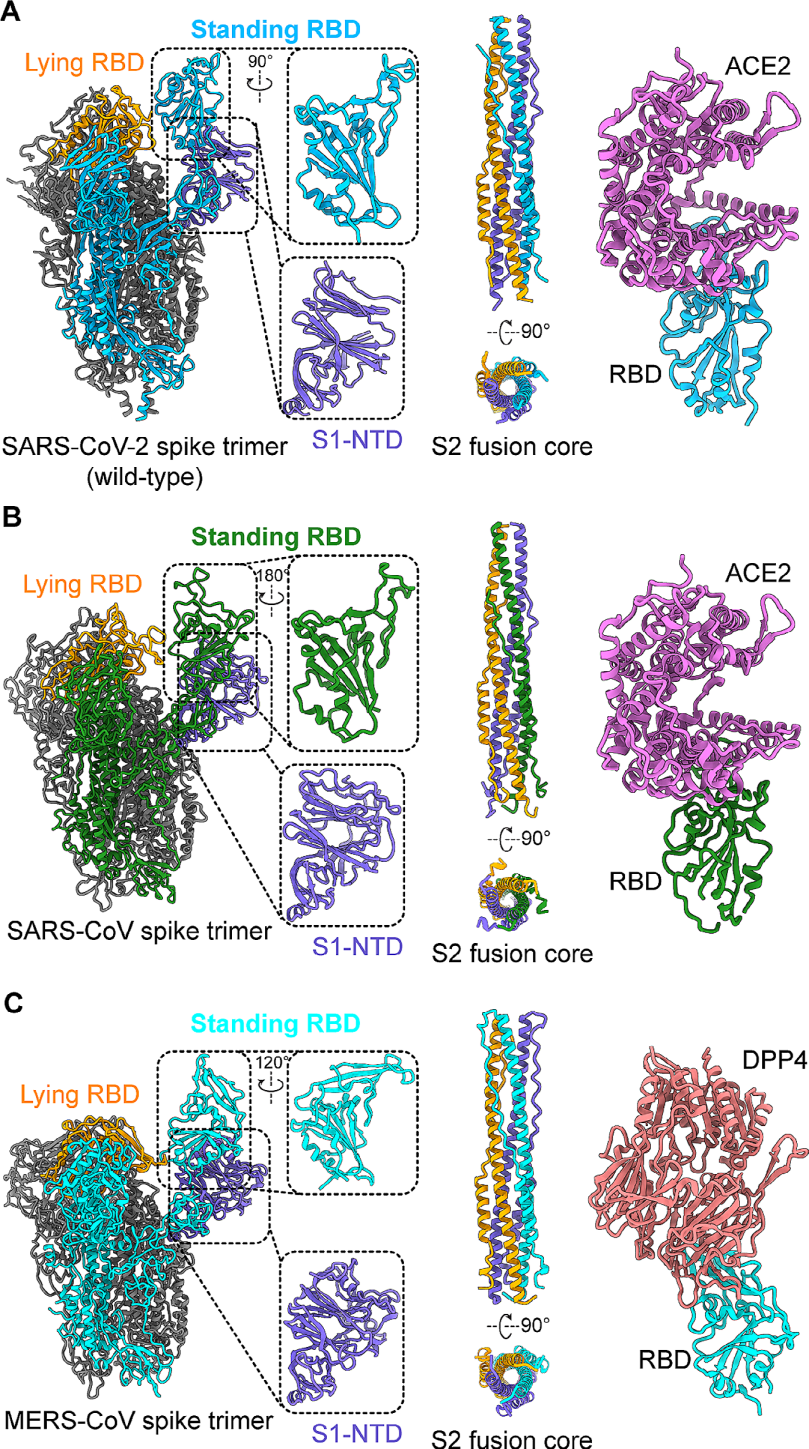
Structural dissection of coronavirus spike proteins. (**A** ) Structures of SARS-CoV-2 spike trimer with close-up views of the S1 N-terminal domain (NTD) and receptor-binding domain (RBD) (PDB 6VYB), the S2 fusion core (PDB 6LXT), and its RBD in complex with receptor angiotensin-converting enzyme 2 (ACE2) (PDB 6M0J). (**B**) Structures of SARS-CoV spike trimer with close-up views of the S1-NTD and RBD (PDB 5 × 5B), the S2 fusion core (PDB 1WYY), and its RBD in complex with receptor ACE2 (PDB 2AJF). (**C**) Structures of MERS-CoV spike trimer with close-up views of the S1-NTD and RBD (PDB 5 × 5 F), the S2 fusion core (PDB 4NJL), and its RBD in complex with receptor dipeptidyl peptidase 4 (DPP4) (PDB 4KR0)

**Fig. 3:**
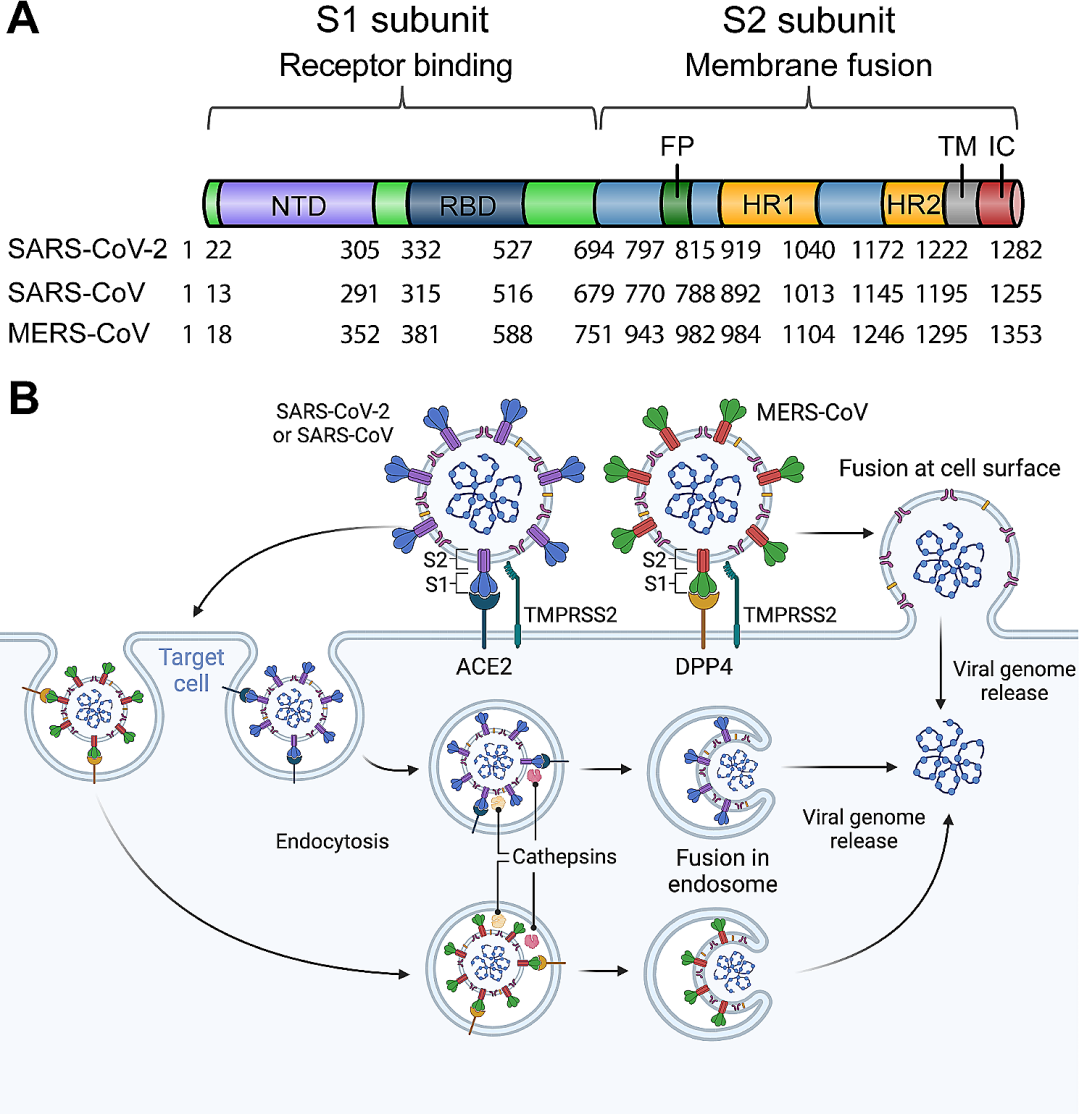
Coronavirus spike-mediated cell entry. (**A**) Schematics of domain organizations of SARS-CoV-2, SARS-CoV, and MERS-CoV spike proteins. The approximate amino acid residue numbers at the domain boundaries are indicated. FP, fusion peptide; HR1 and HR2, heptad region 1 and 2; TM, transmembrane domain; IC, intracellular tail. (**B**) Mechanisms of cell entry of SARS-CoV-2, SARS-CoV, and MERS-CoV. The membrane fusion occurs either at the target cell surface upon the cleavage of spike proteins by host transmembrane serine protease 2 (TMPRSS2), or in endosomes upon the cleavage of spike proteins by endosomal cathepsins

The S protein of SARS-CoV, MERS-CoV, and SARS-CoV-2 mediates viral infection and pathogenesis. It is accountable for both receptor binding and the subsequent membrane fusion and viral entry processes [48–50]. Located on the surface of the virion, the S protein is displayed as a trimer (Fig. 2A-C). The monomeric S protein comprises two critical subunits, named S1 and S2. S1 contains two key components (fragments): the N-terminal domain (NTD) and the RBD [51]. The S2 subunit contains the fusion peptide (FP), heptad repeat 1 and 2 (HR1 and HR2), and the transmembrane region (TM) (Figs. 2 and 3A) [49]. During infection, the virus first binds to a cellular receptor on host cells via the RBD region of the S1 subunit to form an RBD/receptor complex, which then triggers a conformational change in the S protein; thus involves a change from a pre-fusion state to an intermediate state, as well as formation of six-helix bundle (6-HB) structures, resulting in fusion between the viral and cell membranes, mediating the virus to enter host cells (Figs. 2 and 3B) [49, 52].

SARS-CoV-2 and SARS-CoV utilize a different receptor from MERS-CoV for viral binding and entry. The former use angiotensin converting enzyme 2 (ACE2), whereas the latter uses dipeptidyl peptidase 4 (DPP4), for entering host cells (Fig. 3B) [53–56]. Each RBD contains a core region and a receptor-binding motif (RBM) subdomain; the latter contains residues critical for direct interaction with the receptor [51, 54]. Even though the core regions of SARS-CoV-2 and SARS-CoV are structurally similar to that of MERS-CoV, their RBM regions are different, resulting in their use of different receptors [51, 57]. Specifically, the RBM of SARS-CoV covers amino acid (aa) residues 424–494 of the RBD, the RBM of SARS-CoV-2 contains 16 critical residues within aa 417–487 of the RBD, and the RBM of MERS-CoV spans residues 484–567; these regions are essential for binding to the ACE2 or DPP4 receptor to form an RBD/ACE2 complex (for SARS-CoV-2 or SARS-CoV RBD) or an RBD/DPP4 complex (for MERS-CoV RBD) (Fig. 2) [53, 57, 58].

Although the RBDs of SARS-CoV-2 and SARS-CoV bind to human ACE2, their binding affinity is tentatively different. Crystal and cryo-EM structural analyses and binding assays indicate that the SARS-CoV-2 RBD or S protein has higher affinity for human or bat ACE2 than that of SARS-CoV, and different residues within the RBD are critical for stabilizing the binding interaction [54, 59, 60]. The affinity of the SARS-CoV-2 S or its RBD for ACE2 receptor also differs between the wild-type and variants, and some mutations may play an essential role in these differences. For instance, the RBD of SARS-CoV-2 Omicron has lower affinity for human ACE2 than that of the Delta variant, but similar affinity to that of the wild-type [61]. The Omicron S protein has higher affinity than the wild-type S protein for mouse ACE2, but similar affinity for hamster and human ACE2 [62]. Mutations in the RBD (S477N, E484K, and N501Y), which were identified in early or recent variants, are responsible for the RBD/ACE2 interaction [63]. The Omicron RBD adapts to mouse ACE2 before infecting humans; four mutated residues (Q493R, Q498R, N501Y, and Y505H) are responsible for the higher binding affinity for mouse ACE2 than for human ACE2 [64]. Moreover, residues 493 and 496, which demonstrated good adaptation to human ACE2 in earlier variants, but poor adaptation in early Omicron subvariants, again showed good adaptation to human ACE2 in the more recent Omicron subvariants [65].

The S proteins and RBD of SARS-CoV, MERS-CoV, and SARS-CoV-2 contain multiple neutralizing epitopes capable of inducing highly potent neutralizing antibodies; therefore, they are key targets for development of effective therapeutic antibodies and vaccines against CoV infection [2, 7, 66, 67]. A number of crystal and cryo-EM structures of the S proteins or RBDs of these CoVs or SARS-CoV-2 variants, as well as complexes with their respective receptors from human or other species, have been solved (Fig. 2) [2, 59, 68, 69], providing important information that can be used when designing effective counter measures to prevent and treat these viral infections.

### Nanobodies and their application as therapeutic agents

**Fig. 4:**
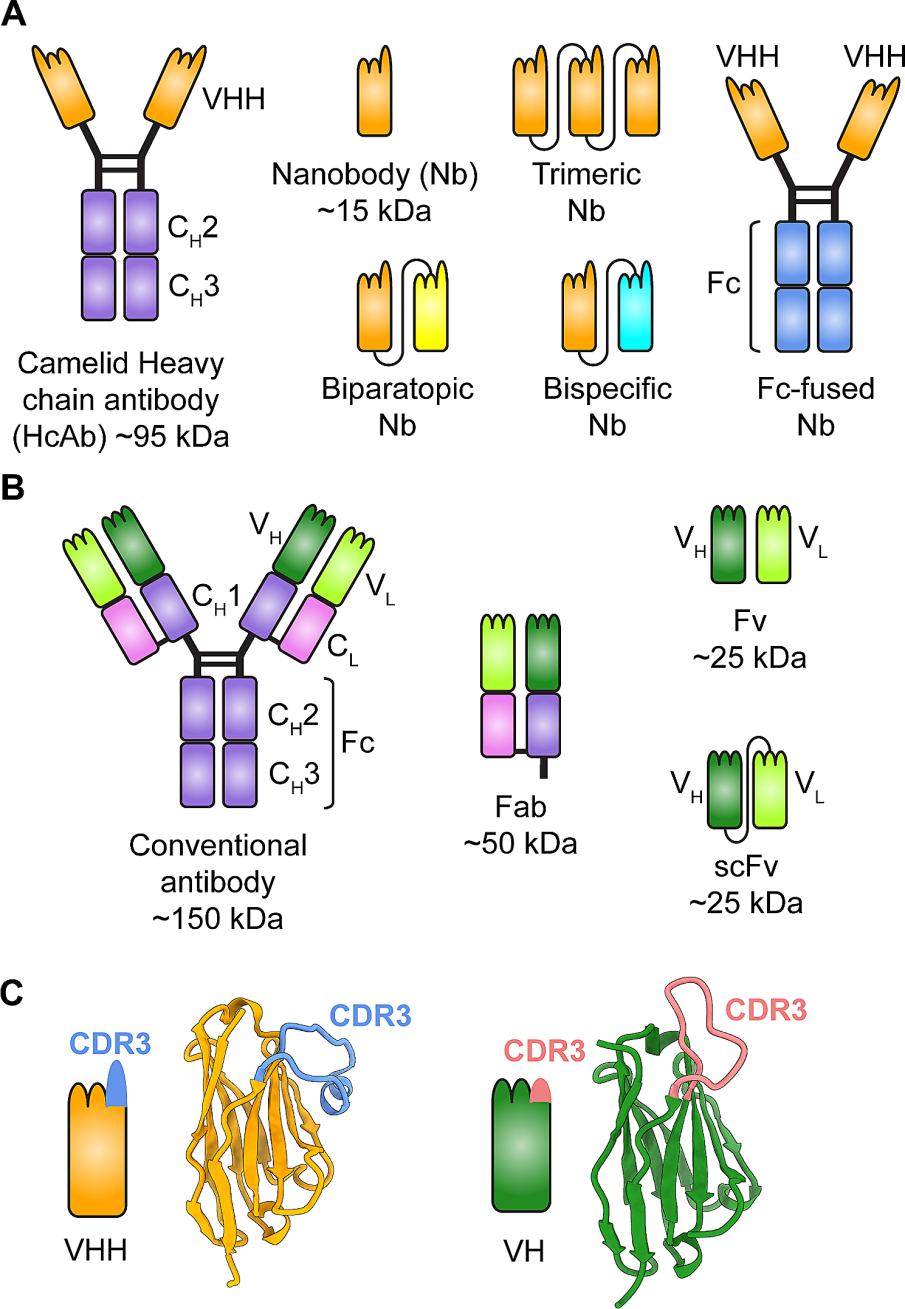
Comparison of nanobodies and conventional antibodies. (**A**) Schematic structures of camelid heavy-chain antibody (HcAb) and variants of nanobodies (Nb). VHH, variable heavy-chain domain of HcAb. (**B**) Schematic structures of conventional antibody and its variants. Fab, fragment antigen-binding; Fv, variable fragment; scFv, single-chain Fv. (**C**) Structural comparison of the variable heavy-chain domain from HcAb (VHH or nanobody) and that from conventional antibody (VH). The complementarity-determining region 3 (CDR3) of VHH and VH are indicated and colored in blue and coral, respectively

Nanobodies, also called natural single-domain antibodies, comprise the antigen-binding region (i.e., VHH) derived from the variable domain of camelid heavy-chain antibodies (HCAbs) (Fig. 4A). Different from conventional antibodies or fragments (i.e., fragment antigen-binding region (Fab) or single-chain variable fragment (scFv)) (Fig. 4B), nanobodies have unique properties, which include small size (~ 15 kDa), strong binding affinity (for target antigens), good tissue penetration, good solubility and stability, and high resistance to extreme conditions, such as low pH and high temperature [70]. What distinguishes nanobodies from the variable heavy chain (VH) of conventional antibodies is the fact that nanobodies have a long complementarity determining region 3 (CDR3), which allows them access to cavities or epitopes unreachable by conventional antibodies (Fig. 4C) [71]. Nanobodies have very low immunogenicity in humans since the VHH sequence is highly homologous with the corresponding region of the human VH chain; if needed, the VHH may be easily humanized by substituting related residues with those from the human VH domain. This minimizes immunogenicity without affecting functionality [71].

Nanobodies can be fused with two or more homologous or heterologous molecules to form dimeric (bispecific or biparatopic) or trimeric multimers to improve their avidity, neutralizing efficiency, and/or protective efficacy (Fig. 4A). Due to their low molecular weight (~ 15, 30, and 45 kDa, respectively, for a monomeric, dimeric, or trimeric nanobody, respectively), which is below the renal filtration threshold (~ 60 kDa), unbound nanobodies can be cleared quickly from the bloodstream through renal elimination [71]. To increase their half-life, nanobodies can be genetically engineered to allow expression as a fusion protein, such as the Fc region of human IgG or an albumin-binding domain [72, 73]. Of note, these nanobody derivatives are still soluble and can be produced efficiently in large quantities in *E.coli* or yeast expression systems, with excellent functionality, thereby allowing scale up and manufacture in large quantities [74].

Because of their unique properties and a variety of advantages over conventional antibodies, nanobodies have been developed rapidly in recent years to prevent and treat viral infections, including those caused by influenza virus, Ebola virus, HIV-1, respiratory syncytial virus, and enterovirus 71 [75–78]. Several nanobodies have proceeded to clinical trials or have been officially approved for treatment of human diseases, such as acquired thrombotic thrombocytopenic purpura, rheumatoid arthritis, plaque psoriasis, solid tumors, and cancer [79–82]. The effective therapeutic efficacy of nanobodies as a treatment for these human diseases brings hope that nanobody therapies can be developed to treat infectious diseases caused by current emerging or future pandemic CoVs.

As described above, the S proteins and RBD fragments of CoVs are key targets for induction of effective antibodies, including nanobodies, against CoV infection. Particularly, SARS-CoV, MERS-CoV, and SARS-CoV-2 are the three major CoVs that have caused outbreaks or pandemics in humans, and all continue to post a threat. The reminder of this review will describe nanobodies respectively targeting SARS-CoV-2, MERS-CoV, and SARS-CoV, focusing on those that target the S protein, including the RBD. We will also discuss their cross-reactive or cross-neutralizing potency (and other properties), and highlight the challenges for development of nanobodies specific for these CoVs. Finally, we will discuss potential strategies for improving the efficiency of nanobodies targeting current dominant and future SARS-CoV-2 variants and pandemic CoVs.

## SARS-CoV-2-specific nanobodies

A variety of nanobodies targeting SARS-CoV-2 have been developed, all of which are derived from immunized animals (i.e., llama, alpaca, sharks, camels, or camelid mice) or synthesized nanobody libraries [83–91]. The nanobody repertoires can be displayed on phages or yeasts, and nanobodies are produced in large quantities using different expression systems, including *E.coli*, yeast, plant, and mammalian cells [92–94]. These nanobodies demonstrate various degrees of neutralizing activity against the SARS-CoV-2 original strain and/or its variants, thereby protecting animals from viral infection. They are delivered to animals through different routes, including intraperitoneal, aerosol, or intranasal, and are considered to be effective and affordable prophylactic and therapeutic agents against SARS-CoV-2 and its variants. The SARS-CoV-2-specific nanobodies are categorized as those targeting the RBD, NTD, other S regions, or other proteins of SARS-CoV-2, as described below (Table 1).

**Table 1:** Representative coronavirus-specific nanobodies

Name	Target	Binding affinity	Neutralizing activity (in vitro)	Protective efficacy (in vivo)	Ref.
**SARS-CoV-2-specific Nbs**					
aRBD-2-5 aRBD-2-7 aRBD-2-5-Fc aRBD-2-7-Fc	RBD	Bind to RBDs of SARS-CoV-2 WT (*K*_*D*_ 0.012–0.25 nM) and variants (Alpha, Beta, Gamma, Delta: *K*_*D*_ 0.005–0.714 nM)	Neutralize infection of SARS-CoV-2 live WT (IC_50_ 0.043–0.111 nM) and variants (Alpha, Beta, Delta, Omicron-BA.1: IC_50_ 0.027–0.13 nM) or pseudotyped variants (Omicron-BA.1, BA.2: IC_50_ 0.02–0.077 nM)	aRBD-2-5-Fc protects K18-hACE2 from SARS2 WT strain, BALB/c mice from MA strain, and hamsters from SARS-CoV-2 Omicron-BA.1 variant infection	[95, 99]
NM1267 NM1268	RBD	Bind to RBDs of SARS-CoV-2 variants (Alpha, Beta, Gamma, Delta) (*K*_*D*_ 0.056–0.764 nM)	Neutralize infection of SARS-CoV-2 variants (Beta, Delta: IC_50_ 0.67–52.55 nM)	Protect K18-hACE2 mice from SARS-CoV-2 Beta and Delta variant infection	[100]
Nb15 Nb56	RBD	Bind to RBD of SARS-CoV-2 WT (*K*_*D*_ 0.164–8.15 nM for monomers or trimers)	Neutralize infection of SARS-CoV-2 pseudotyped WT (IC_50_ 0.055-0.9 nM for monomers, dimers, or trimers) or live WT and variants (Alpha, Beta, Gamma) (IC_50_ 0.003–0.106 nM)	N/A	[83]
S2-3-IgA2m2	RBD	Binds to RBDs of SARS-CoV-2 WT (EC_50_ 0.042 nM) and variants (Omicron-XBB, BA.1, BA.2: *K*_*D*_ 0.001–0.003 nM)	Neutralizes infection of SARS-CoV-2 pseudotyped WT (IC_50_ 0.017 nM) and Omicron-XBB variant (IC_50_ 0.024 nM), or live WT and variants (Delta, Omicron-BA.1, BA.2, BA.5) (IC_50_ 0.006–0.04 nM)	Protects hamsters from SARS-CoV-2 Omicron-BA.5 variant infection	[104]
W25-Fc	RBD or S	Binds to RBD of SARS-CoV-2 WT and S of WT and variants (Alpha, Beta, Gamma, Delta, Omicron-BA.1) (*K*_*D*_ <0.001–11.4 nM)	Neutralizes infection of live SARS-CoV-2 WT and variants (Alpha, Beta, Gamma, Omicron-BA.1, BA.2) (IC_50_ 0.38–9.01 nM)	Protects K18-hACE2 mice from SARS-CoV-2 Beta variant infection	[98]
ShAb01	RBD	Binds to RBDs of SARS-CoV-2 WT and variants (Alpha, Beta, Delta) (*K*_*D*_ 38.9–70.8 nM)	Neutralizes infection of pseudotyped SARS-CoV-2 WT and variants (Alpha, Beta, Delta) (IC_50_ 188–619 ng/ml)	Protects K18-hACE2 mice from SARS-CoV-2 WT strain infection	[86]
TN^T^DNGR-1	RBD	Binds to RBD of SARS-CoV-2 (B.1 strain)	Neutralizes infection of pseudotyped SARS-CoV-2 variants (Beta, Delta) (IC_50_ 0.037–0.043 nM)	Protects K18-hACE2 mice from SARS-CoV-2 B.1 strain infection	[106]
Re32D03	RBD	Binds to RBDs of SARS-CoV-2 WT and variants (Beta, Gamma, Delta, Omicron-BA.5) (*K*_*D*_ <0.01-20 nM)	Neutralizes infection of pseudotyped SARS-CoV-2 WT and variants (Alpha, Delta)	Protects hamsters from SARS-CoV-2 WT strain infection	[107]
R14	RBD	Binds to RBDs of SARS-CoV-2 Beta and Gamma variants (*K*_*D*_ 0.02–0.03 nM)	Neutralizes infection of SARS-CoV-2 live WT (IC_50_ 1.3 nM) and pseudotyped WT and variants (Alpha, Beta, Gamma, Delta) (IC_50_ 0.16–0.6 nM)	Protects Ad5-hACE2 mice from SARS-CoV-2 WT strain infection	[108]
Nanosota-3 A-Fc	RBD	Binds to RBD of SARS-CoV-2 WT (*K*_*D*_ 4.55 nM)	Neutralizes infection of SARS-CoV-2 pseudotyped WT or variants (Alpha, Omicron-BA.1) (IC_50_ 1.2–5.7 ng/ml) and live Omicron-BA.1 variant (IC_50_ 2.3 ng/ml)	Protects K18-hACE2 and/or BALB/c mice from SARS-CoV-2 Omicron-BA.1 variant infection	[94]
2-3-Fc	RBD	Binds to RBDs of SARS-CoV-2 Omicron variants (BA.1, BA.2, BA.5) (*K*_*D*_ 0.24–0.35 nM)	Neutralizes infection of SARS-CoV-2 live WT or variants (Beta, Delta, Omicron-B.1) (IC_50_ 0.024–0.095 nM) and pseudotyped Omicron variants (BA.1, BA.2, BA.5) (IC_50_ 0.037–0.074 nM)	Protects hamsters from SARS-CoV-2 Omicron-BA.1 variant infection	[109]
SR01 SR02 SR01-Fc SR02-Fc	NTD	Binds to NTDs of SARS-CoV-2 WT and variants (Alpha, Beta) (*K*_*D*_ 0.06–0.59 nM)	Neutralize infection of SARS-CoV-2 WT and variants (Alpha, Beta, Omicron) (IC_50_ 3.77–300 nM)	SR01-Fc partially protects hamsters from SARS-CoV-2 WT strain infection	[110]
S2A3 S2A3-Fc	S2	Binds to S2 of SARS-CoV-2 WT and variants (Alpha, Beta) (*K*_*D*_ 0.56–2.18 nM)	Neutralize infection of SARS-CoV-2 WT and variants (Alpha, Beta, Omicron) (IC_50_ 5.36-54 nM)	S2A3-Fc partially protects hamsters from SARS-CoV-2 WT strain infection	[110]
S2A9 S2A9-Fc	S2	Binds to S2 of SARS-CoV-2 (*K*_*D*_ 270 nM)	Neutralize infection of pseudotyped SARS-CoV-2 WT and variants (Alpha, Beta, Gamma, Delta, Omicron-BA.1, BA.2, BA.4/BA.5) (IC_50_ 1711–4134 nM for S2A9; IC_50_ 113.7–1854 nM for S2A9-Fc); S2A9 neutralizes infection of several live SARS-CoV-2 variants (IC_50_ 186–2136 nM)	N/A	[87]
**SARS-CoV-specific Nbs**					
VHH-1 VHH-6 VHH-44 -72	S or RBD	VHH-72 binds to RBD of SARS-CoV (*K*_*D*_ 1.2 nM)	Neutralize infection of pseudotyped SARS-CoV (IC_50_ 9 nM for VHH-72; 355 nM for VHH-44)	N/A	[114]
S14	RBD	Binds to RBD of SARS-CoV (*K*_*D*_ 0.143 nM)	Neutralizes infection of pseudotyped SARS-CoV (IC_50_ 4.93 ng/ml)	N/A	[115]
**MERS-CoV-specific Nbs**					
VHH-55 VHH-12 VHH-34 VHH-40	S1 or RBD	VHH-55 binds to RBD of MERS-CoV (*K*_*D*_ 0.079 nM)	Neutralize infection of pseudotyped MERS-CoV (IC_50_ 0.9-193.3 nM)	N/A	[114]
VHH-1 VHH-4 VHH-83 VHH-101	RBD	Bind to S of MERS-CoV (*K*_*D*_ 0.1-1 nM)	Neutralize infection of live MERS-CoV (IC_50_ 0.093-0.8 nM)	Fc of VHH-83 (HCAb-83) protects K18-hDPP4 mice from MERS-CoV infection	[116]
NbMS10-Fc	RBD	Binds to RBD of MERS-CoV (*K*_*D*_ 0.35 nM)	Neutralizes infection of live MERS-CoV (IC_50_ 2.33 µg/ml)	Protects hDPP4-Tg mice from MERS-CoV infection	[118]
**Cross-reactive or cross-neutralizing Nbs**					
VHH-72 VHH-72-Fc	RBDs	VHH-72 binds to RBDs of SARS-CoV (*K*_*D*_ 1.2 nM), SARS-CoV-2 (*K*_*D*_ 39 nM), and W1V1 (*K*_*D*_ 7.4 nM)	VHH-72-Fc neutralizes infection of live SARS-CoV-2 WT (IC_50_ 786 ng/ml); its engineered mutants improve this neutralizing activity (IC_50_ 0.31–1.91 ng/ml)	N/A	[114, 119]
10B8-Fc	RBDs	Binds to S of SARS-CoV-2 WT or variants (*K*_*D*_ 0.03–2.81 nM), and SARS-CoV (EC_50_ 0.14 nM)	Neutralizes infection of SARS-CoV-2 WT or variants (Alpha, Beta, Gamma, Omicron) (IC_50_ 0.21–4.1 nM), and SARS-CoV (IC_50_ 0.09 nM)	N/A	[101]
aSA3-Fc	RBDs	Binds to RBDs of SARS-CoV-2 WT (*K*_*D*_ 0.01 nM) or variants, and SARS-CoV (*K*_*D*_ 0.085 nM)	Neutralizes infection of pseudotyped SARS-CoV-2 WT (IC_50_ 0.36 nM) or SARS-CoV (IC_50_ 0.1 nM), live SARS-CoV-2 WT and variants (Beta, Delta, Omicron-BA.1) (IC_50_ 0.17–0.5 nM), and live W1V1 (IC_50_ 1.04 nM)	N/A	[109]
3-2A2-4	RBD	Binds to SARS-CoV-2 RBD	Neutralizes infection of live SARS-CoV-2 WT and variants (Alpha, Beta, Delta, Omicron) (IC_50_ 98–130 ng/ml), and pseudotyped SARS-CoV (IC_50_ 16 ng/ml), pangolin-CoV (GD/GX, IC_50_ 34–500 ng/ml), or bat-CoVs (WIV16, RaTG13-T372A, IC_50_ 37–50 ng/ml)	Protects K18-hACE2 mice from SARS-CoV-2 Delta and Omicron (BA.1) variant infection	[84]
S43	RBDs	Binds to RBDs of SARS-CoV-2 WT (*K*_*D*_ 0.18 nM) or variants (Alpha, Beta, Gamma, Delta: *K*_*D*_ 0.06–0.5 nM), SARS-CoV, RaTG13, and GD/1/2019 (*K*_*D*_ 0.19–0.26 nM)	Neutralizes infection of SARS-CoV-2 live WT (IC_50_ 40.7 nM) and pseudotyped WT or variants (Alpha, Beta, Gamma, Delta) (IC_50_ 2.8–13.1 nM)	Protects Ad5-hACE2-transduced mice from SARS-CoV-2 WT strain infection	[108]
Nanosota-4 A-Fc	SARS-CoV-2 RBD	Binds to RBD of SARS-CoV-2 WT (*K*_*D*_ 0.184 nM)	Neutralizes infection of pseudotyped SARS-CoV-2 WT or variants (Alpha, Delta), SARS-CoV, and bat-CoV (IC_50_ 8.8–24 ng/ml)	N/A	[94]

*Note* CoV, coronavirus; hACE2, human angiotensin converting enzyme 2; hDPP4, human dipeptidyl peptidase 4; IC_50_, half-maximal inhibitory concentration; EC_50_, half maximal effective concentration; *K*_*D*_, d*issociation* equilibrium constant; MA, mouse-adapted; MERS-CoV, Middle East respiratory symptom-CoV; N/A, not available; Nb, nanobodies; NTD, N-terminal domain; RBD, receptor-binding domain; S, spike; SARS-CoV-2, severe acute respiratory symptom CoV-2; Tg, transgenic; WT, wild-type

### SARS-CoV-2 RBD-targeting nanobodies

Most of the currently developed nanobodies target the RBD of the original SARS-CoV-2 S protein, although a number of these also target the RBD of SARS-CoV-2 variants. These nanobodies demonstrated effective neutralizing activity against the original strain and/or its variants of SARS-CoV-2, including Omicron subvariants, with various potencies (Table 1).

#### Major categories based on mechanisms of action

SARS-CoV-2 RBD-specific nanobodies can be categorized as those that target the receptor ACE2-binding sites to completely, or partially, compete with the ACE2 for binding to the RBD (i.e., aRBD-2, aRBD-5, aRBD-7, and NB1B5), or those that target conserved RBD epitopes in cryptic epitopes or epitopes outside of the ACE2-binding sites (these nanobodies, which do not compete for RBD-ACE2 binding, include 3-2A2-4, NB1C6, Nb12, and Nb30) [83, 84, 95, 96]. Notably, nanobodies recognizing conserved RBD epitopes may maintain binding to the RBD of SARS-CoV-2 variants, whereas those targeting the less-conserved RBD epitopes tend to show reduced binding affinity for the RBD of different variants [95]. Some nanobodies, such as VH103, recognize RBD-enhancing linear epitopes, whereas others such as W25 stimulate S-dependent cell-cell fusion [97, 98].

#### Antigens for screening nanobodies

Most SARS-CoV-2 RBD-targeting nanobodies are generated through screening of phage display libraries using full-length S protein or RBD fragment [99–101]. Other nanobodies can be generated by using short peptide sequences from the RBD or S protein as epitopes. For example, a 9-amino acid peptide from the RBD (residues 486–494 of S protein) was used to identify nanobody N1.2, which blocks SARS-CoV-2 S protein-mediated pseudovirus entry; the bivalent version of this nanobody shows improved neutralizing activity against the pseudotyped SARS-CoV-2 original strain and the Omicron-BA1 variant [102].

#### Bispecific, biparatopic, or multivalent nanobodies

These nanobodies exhibit affinity for the RBD, resulting in an increased ability to neutralize SARS-CoV-2 wild-type stain or variants [95, 103]. Hetero-bivalent nanobodies aRBD-2-5 and aRBD-2-7, which were generated by respectively fusing two RBD-targeting nanobodies with non-overlapping epitopes, show increased affinity for the RBD, as well as higher neutralizing activity [99]. A bispecific tetra-nanobody immunoglobulin, which was generated by replacing the VH and VL domains of a conventional antibody with two different nanobodies, neutralizes SARS-CoV-2 variants [103]. Biparatopic nanobodies NM1267 and NM1268, which recognizes a conserved epitope outside the RBD-ACE2 binding interface, and two different epitopes inside this interface, bind strongly to the RBDs of Alpha, Beta, Gamma, or Delta variants and neutralized the Beta and Delta variants [100]. Biparatopic or multivalent formats of NB1C6 and NB1B5 show even higher binding affinity and neutralizing activity against the Omicron variant, significantly inhibiting viral escape [96]. Different from their monomeric formats, homotrimer Nb15 and Nb56 maintain potent binding affinity and neutralize SARS-CoV-2 pseudoviruses containing mutations (E484K and/or N501Y) in the SARS-CoV-2 RBD [83].

#### Fusion-tagged nanobodies

SARS-CoV-2 RBD-targeting monomeric or dimeric nanobodies can be further fused to the Fc of human IgG, IgA, or other tags to improve their binding affinity and neutralizing activity [89, 95, 104]. G12 × 3-Fc has high affinity for the original RBD, and maintains effective binding to the SARS-CoV-2 Delta and Beta variant RBDs, with effective or decreased neutralizing ability against live Delta and Omicron-BA.1 variants, respectively [89]. Fc-fused aRBD-2-5 and aRBD-2-7 hetero-bivalent nanobodies show higher affinity than each individual Fc-tagged nanobodies for the RBDs of wild-type, Alpha, and Delta variants, and improve neutralizing activity against the pseudotyped or live SARS-CoV-2 original strain and variants (Alpha, Beta, Gamma, Delta, and Omicron-BA.1 and BA.2) [95]. A nanobody fused to secretary IgA (S2-3-IgA2m2) neutralizes SARS-CoV-2 variants (XBB, BQ.1.1) more potently than when fused to IgG [104].

#### Protective efficacy and routes of delivery

A variety of SARS-CoV-2 RBD-targeting nanobodies have been proven to protect against SARS-CoV-2 infection in different animal models, including mice (i.e., K18-hACE2 transgenic or BALB/c) and hamsters, and one of these, XVR011, has been proceeded to human clinical trials [95, 98, 105]. The majority of these nanobodies are delivered to animals via intraperitoneal injection. Some are fused to human IgG Fc to improve their prophylactic protective potency and/or therapeutic activity against SARS-CoV-2 original strain and/or its variants, resulting in reduced viral load, lower disease burden, and lower mortality [98]. For example, Fc-fused aRBD-2-5 nanobodies derived from alpacas prophylactically protect K18-hACE2 mice or BALB/c mice against the SARS-CoV-2 original and mouse-adapted strains, respectively, and prophylactically and therapeutically protect hamsters from the Omicron BA.1 variant [95]. Alpaca-derived and Fc-fused W25-Fc provide K18-hACE2 mice with prophylactic and therapeutic protection against Beta variant-induced death and weight loss, with a decreased viral load in the nasal turbinate and lung [98]. Fc-fused nanobodies, such as ShAb01 derived from sharks, also provide K18-hACE2 mice with prophylactic protection against SARS-CoV-2 wild-type, with delayed weight loss, increased survival, and reduced viral titers [86]. Other nanobodies can be fused to DNGR-1-expressing dendritic cells for selective targeting. For example, TN^T^DNGR-1, which was generated by fusing a SARS-CoV-2 RBD-specific biparatopic nanobody (VHH)-based trimerbody (TN^T^) with a DNGR-1-specific scFV, provides K18-hACE2 mice with therapeutic protection from challenge with lethal SARS-CoV-2 (B.1. strain), with a reduced viral load in the lungs [106].

SARS-CoV-2 RBD-targeting nanobodies also provide effective protection after inhalation or intranasal delivery [94, 104, 107, 108]. The aerosol-formulated Re32D03 therapeutically or prophylactically protects hamsters from infection by SARS-CoV-2 (prototypic strain), with a decreased viral load, and less weight loss [107]. Aerosol delivery of R14 prophylactically protects Ad5-hACE2-transduced BALB/c mice from infection by prototypic SARS-CoV-2, with reduced viral RNA levels in the lungs [108]. Intranasal delivery of NM1267 and NM1268 biparatopic nanobodies provides K18-hACE2 mice with prophylactic protection from disease progression and mortality caused by Beta and Delta variants [100]. In addition to its therapeutic efficacy against Omicron BA.1 in intraperitoneal-injected K18-hACE2 transgenic and wild-type BALB/c mice, Nanosota-3 A-Fc protects BALB/c mice from this variant when delivered via the intranasal route, with significant reduction of viral titers in the lungs [94]. 2-3-Fc, produced by fusing aSA3-Fc with aRBD-2 and delivered via the intranasal route, prophylactically protects hamsters against challenge with Omicron BA.1, showing reduced viral RNA levels but without viral titers being detected in the trachea and lungs [109]. Intranasal delivery of sIgA-fused S2-3-IgA2m2 protects hamsters from infection with Omicron BA.5, showing a reduced viral load and clearance of the virus from the trachea and lungs [104].

#### Crystal or cryo-EM structures

**Fig. 5:**
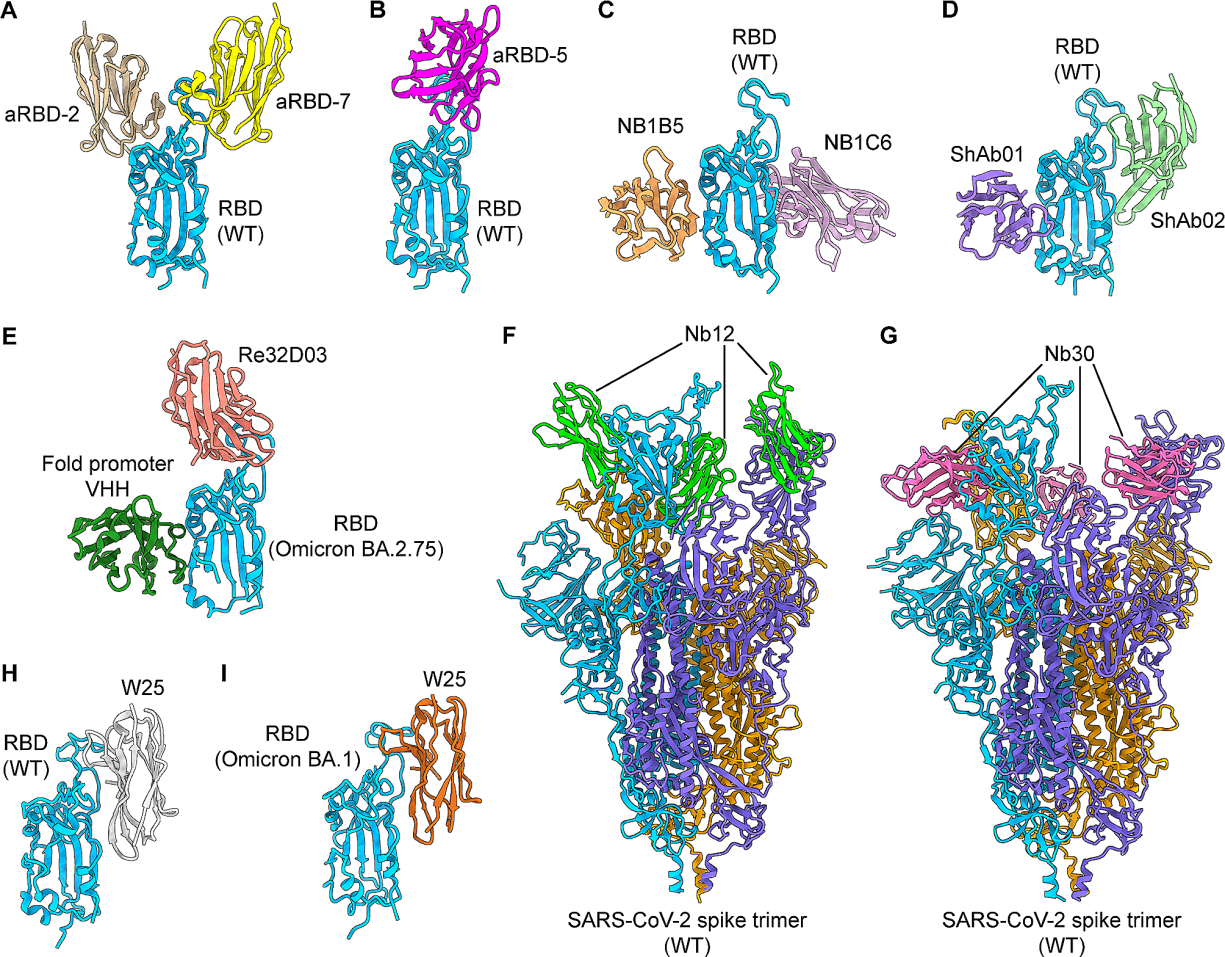
Structures of nanobodies targeting SARS-CoV-2 spike protein or its RBD. (**A**) Crystal structure of nanobodies aRBD-2 and aRBD-7 in complex with SARS-CoV-2 RBD (PDB 7FH0). (**B**) Crystal structure of nanobody aRBD-5 in complex with SARS-CoV-2 RBD (PDB 7VOA). (**C**) Crystal structure of nanobodies NB1B5 and NB1C6 in complex with SARS-CoV-2 RBD (PDB 8HR2). (**D**) Crystal structure of nanobodies ShAb01 and ShAb02 in complex with SARS-CoV-2 RBD (PDB 7S83). (**E**) Crystal structure of nanobody Re32D03 in complex with SARS-CoV-2 Omicron BA.2.75 variant RBD (PDB 8Q94). (**F**) Cryo-EM structure of nanobody Nb12 in complex with SARS-CoV-2 spike trimer (PDB 7MY3). (**G**) Cryo-EM structure of nanobody Nb30 in complex with SARS-CoV-2 spike trimer (PDB 7MY2). (**H**) Local structural model of the cryo-EM structure of nanobody W25 in complex with the RBD of SARS-CoV-2 spike (PDB 8BEV). (**I**) Local structural model of the cryo-EM structure of nanobody W25 in complex with the RBD of SARS-CoV-2 Omicron BA.1 variant spike (PDB 8BGG). WT, wild-type

The crystal and cryo-EM structures of a number of SARS-CoV-2 RBD-targeting nanobodies (such as aRBD-2, aRBD-5, aRBD-7, NB1B5, NB1C6, ShAb01, ShAb02, Re32D03, W25, Nb12, and Nb30) complexed with the RBD or S protein of SARS-CoV-2 original strain or its variants have been solved (Fig. 5A-I) [83, 86, 95, 96, 98, 107], enabling identification of epitopes recognized by nanobodies with potent neutralizing ability, as well as elucidation of their mechanism(s) of neutralization. These structures may explain how nanobodies bind to the S protein or RBD, hereby blocking binding to the ACE2 receptor, or tolerating to escape mutant variants [107]. The structures of these nanobodies provide valuable tools for rational design of effective SARS-CoV-2 therapeutic agents that target the RBD or S protein.

### SARS-CoV-2 NTD-targeting nanobodies

The NTD of SARS-CoV-2 serves as an alternative therapeutic target, but nanobodies targeting this region generally show much less neutralizing activity than those specific for the RBD of SARS-CoV-2 (Table 1). 19B8, which was obtained from an immunized llama, targets the SARS-CoV-2 NTD region [101]. The neutralizing activity of a Fc version of this nanobody against a pseudotyped SARS-CoV-2 prototypic strain (IC_50_ > 50 nM) was much less than that of Fc-fused RBD-targeting nanobodies screened from the same library (i.e., 11F8, 9B2, or 1A11: IC_50_ < 2 nM) [101]. Other llama-derived, SARS-CoV-2 NTD-targeting nanobodies SR01 and SR02 neutralize SARS-CoV-2 prototypic strain and variants, including Alpha, Beta, and Omicron, with a neutralizing potency (IC_50_) ranging from 3.77 to 300 nM for the Fc fusion versions [110].

### Nanobodies targeting other S regions of SARS-CoV-2

The S2 region of SARS-CoV-2 is an additional target for development of therapeutic antibodies, and several nanobodies targeting this region have been generated from immunized llamas and sharks (Table 1) [87, 110]. The llama-derived S2A3 nanobody targets the S2 of SARS-CoV-2, and its Fc format neutralizes infection by the SARS-CoV-2 wild-type strain and its variants (i.e., Alpha, Beta, and Omicron) with different potency (IC_50_ ranging from 5.36 to 54 nM) [110]. Shark-derived S2A9 targets the SARS-CoV-2 S2 and shows broadly neutralizing activity against pseudotyped or authentic SARS-CoV-2 original strains and variants, including Alpha, Beta, Gamma, and Omicron subvariants (i.e., BA.1, BA.2, BA.4, BA.5) [87]. The Fc format of this nanobody shows increased neutralizing potency against several pseudotyped variants, but has lost neutralizing activity against some authentic variants [87].

### Nanobodies targeting other SARS-CoV-2 proteins

Nanobodies targeting other structural proteins (such as NP) and NSPs (such as PLpro) of SARS-CoV-2 have been developed [111, 112]. Although these nanobodies might not demonstrate neutralizing activity or protective efficacy against viral infection, they can be used to develop immunoassays and reagents to detect SARS-CoV-2 proteins, or inhibit protease activity. For example, SARS-CoV-2 NP-targeting nanobodies have been used in an ELISA to detect NP protein, and a chimeric nanobody developed by fusing this nanobody with NanoLuc luciferase effectively increases the sensitivity for clinical diagnosis [111]. Nanobody-displayed whole-cell biosensors show dramatically improved sensitivity for detecting SARS-CoV-2 variant S proteins, having the potential for efficient detection of future SARS-CoV-2 variants [113]. Finally, SARS-CoV-2 PLpro-targeting NB1A1 and NB1F7 nanobodies significantly inhibit PLpro activity in enzyme activity experiments [112].

## SARS-CoV-specific nanobodies

Similar to SARS-CoV-2, SARS-CoV also causes severe respiratory syndrome and utilizes ACE2 as its cellular receptor. Unlike nanobodies targeting SARS-CoV-2, only a few nanobodies target SARS-CoV directly (described below) (Table 1). Other SARS-CoV-specific nanobodies, including those targeting the RBD, show cross-reactivity and/or cross-neutralizing activity against both SARS-CoV-2 and SARS-CoV (which are described in subsequent sections) (Table 1).

**Fig. 6:**
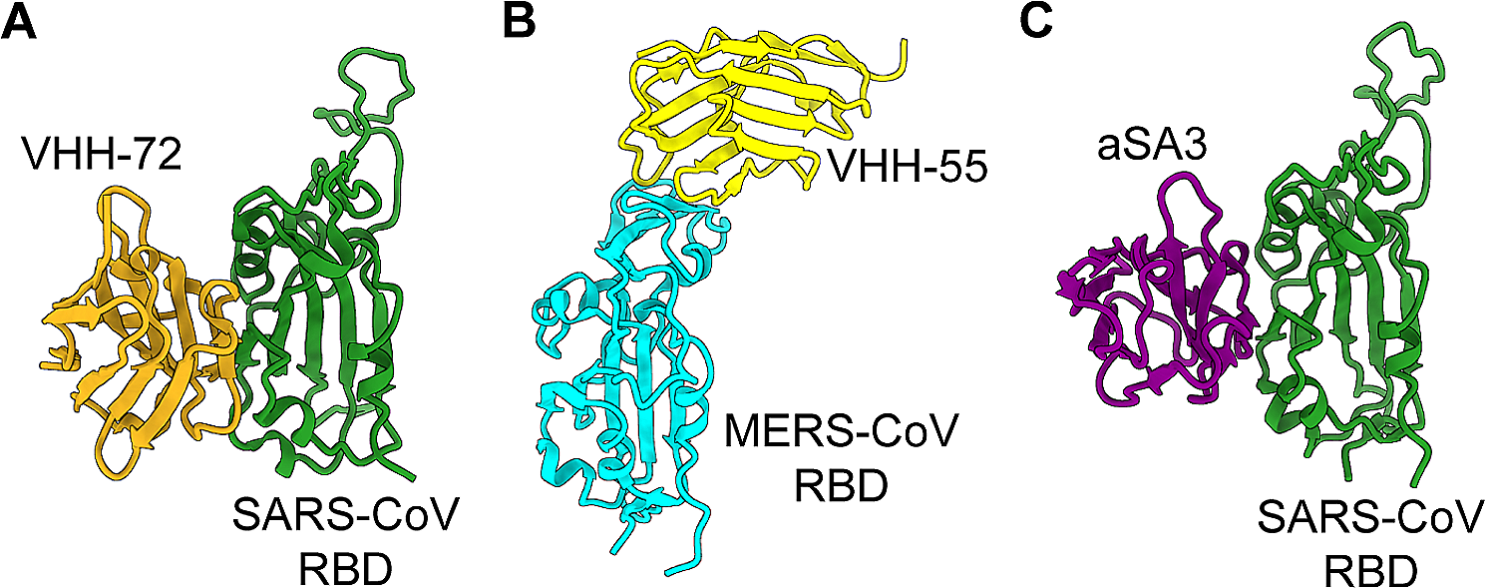
Structures of nanobodies targeting the RBDs of other human coronavirus spikes. (**A**) Crystal structure of nanobody VHH-72 in complex with SARS-CoV RBD (PDB 6WAQ). (**B**) Crystal structure of nanobody VHH-55 in complex with MERS-CoV RBD (PDB 6WAR). (**C**) Crystal structure of nanobody aSA3 in complex with SARS-CoV RBD (PDB 7 × 4I)

Sequential immunization of llamas with SARS-CoV and MERS-CoV S proteins resulted in identification of VHH-72, VHH-1, and VHH-6, all of which show high binding affinity for the SARS-CoV S protein; VHH-72 and another nanobody, VHH-44, show neutralizing activity against pseudotyped SARS-CoV infection (IC_50_: 9 and 355 nM, respectively) [114]. The crystal structure of VHH-72 in complex with SARS-CoV RBD demonstrates that this nanobody binds to SARS-CoV RBD but does not interact with the binding of RBD to the ACE2 receptor (Fig. 6A) [114]. An alpaca-derived phage display library was used to generate S14, which targets the SARS-CoV RBD and neutralizes pseudotyped SARS-CoV, blocking binding of the RBD to the ACE2 receptor [115].

## MERS-CoV-specific nanobodies

Different from SARS-CoV-2 and SARS-CoV, MERS-CoV uses DPP4 to initiate the viral entry process. Several nanobodies targeting MERS-CoV RBD have been identified by screening phage display libraries from immunized llamas, camels, or alpacas; these nanobodies (described below) block binding between the RBD and DPP4, neutralize MERS-CoV infection, and/or protect animals from virus challenge (Table 1) [114, 116–118]. A few nanobodies cross-react with the MERS-CoV S2 region of the S protein (which are described in subsequent sections).

Using the sequential llama immunization strategy described above, VHH-55, VHH-12, VHH-34, and VHH-40, all of which have high binding affinity for MERS-CoV S1 or RBD protein and neutralize pseudotyped MERS-CoV infection (IC_50_: 0.9–193.3 nM), were selected [114]. Among them, VHH-55 binds directly to the MERS-CoV RBD, interrupting its binding to the DPP4 receptor (as shown by the crystal structure of the complex) (Fig. 6B) [114]. Immunization of dromedary camels with a modified vaccinia virus encoding the MERS-CoV S protein, followed by challenge with MERS-CoV, led to the isolation of VHH-1, VHH-4, VHH-83, and VHH-101, all of which target the MERS-CoV S protein RBD with high affinity, and neutralize MERS-CoV infection (IC_50_: 0.093-0.8 nM) [116]. Unlike VHH-83, the Fc version (HCAb-83; 0.2 mg/mouse) prophylactically protected K18-hDPP4 transgenic mice from lethal challenge with MERS-CoV, showing no evidence of weight loss, death, or pathological changes in the lungs [116]. NbMS10, which was derived from an immunized alpaca, binds to the MERS-CoV RBD [117, 118]. The homotrimer format of this nanobody improves RBD-binding affinity significantly; it also shows enhanced inhibition of RBD-DPP4 binding, and increased neutralizing activity against multiple pseudotyped MERS-CoV strains (IC_50_: 0.01–0.61 nM) [117]. Fc-fused NbMS10 (NbMS10-Fc) neutralizes MERS-CoV infection, and prophylactically and therapeutically (10 mg/kg of weight) protects hDPP4 transgenic mice from lethal challenge with MERS-CoV (all mice survived) [118].

## Cross-reactive or cross-neutralizing potency and other properties

### Cross-reactive or cross-neutralizing potency of anti-coronavirus nanobodies

Several nanobodies possess cross-reactivity with the RBDs of SARS-CoV and SARS-CoV-2, and cross-neutralize SARS-CoV-2 wild-type and variants, as well as SARS-CoV (Table 1). Deep mutational engineering and yeast surface display was used to develop VHH-72 Nb, which originally only bound specifically to the SARS-CoV RBD; the engineered nanobodies cross-react with the SARS-CoV-2 RBD, and their fusion proteins with Fc further increase the binding affinity for SARS-CoV-2 variants (Gamma and Delta) and neutralizing activity against SARS-CoV and SARS-CoV-2 infections [114, 119]. Shark-derived, SARS-CoV-2 RBD-targeting nanobodies ShAb01 and ShAb02 bind effectively to SARS-CoV-2 variants and neutralize pseudotyped SARS-CoV-2 original strain and its variants (Alpha, Beta, Delta, Omicron BA.1 or BA.5), as well as SARS-CoV [86]. Llama-derived 10B8 and 1E4 neutralize SARS-CoV-2 and SARS-CoV, and the bivalent 10B8 shows increased neutralization potency [101]. Alpaca-derived aSA3 binds tightly to the RBDs of SARS-CoV and SARS-CoV-2, effectively neutralizing SARS-CoV and SARS-CoV-2 pseudoviruses [109]. A Fc-fused version of aSA3 has increased RBD-binding affinity, and neutralizing activity against the SARS-CoV and SARS-CoV-2 original strain and variants (Beta, Delta, Omicron BA.1), but with significantly less potency against Omicron BA.2 and BA.5; this may be due to the fact that aSA3 blocks RBD-ACE2 binding less efficiently [109].

Several nanobodies may bind to other sarbecovirus RBDs or neutralize these CoVs in addition to SARS-CoV-2 and/or SARS-CoV (Table 1). Nb12 and Nb30 bind to the RBDs of SARS-CoV-2, SARS-CoV, and bat CoV (WIV16), and neutralize the respective pseudoviruses [83]. Alpaca-derived, RBD-targeting 3-2A2-4 neutralizes the SARS-CoV-2 original strain and its variants (i.e., Alpha, Beta, Delta, Omicron BA.1, BA.2 and BA.4/5), as well as SARS-CoV and other sarbecoviruses (i.e., pangolin and bat CoVs), thereby protecting K18-hACE2 mice from infection by the SARS-CoV-2 Delta and Omicron (BA.1) variants [84]. S43 shows neutralizing activity against infection by the SARS-CoV-2 prototypic stain and the Alpha, Beta, and Delta variants; it also binds to the RBDs of other sarbecoviruses such as SARS-CoV, RaTG13, and GD/1/2019 [108]. Alpaca-derived Nanosota-3 A-Fc neutralizes pseudotyped SARS-CoV-2 Alpha, Delta, and Omicron (BA.1) variants, Nanosota-3B-Fc neutralizes the Alpha and Omicron (BA.1, BA.5, and XBB.1.5) variants, and Nanosota-4 A-Fc cross-neutralizes pseudotyped SARS-CoV and a SARS-like bat-CoV (Rs3367); all of these nanobodies cross-neutralize a SARS2-like bat-CoV (BANAL236) with different potencies [94].

Nanobodies with cross reactivity against all three highly pathogenic CoVs are rare, but some have been identified. Such nanobodies appear to bind to the conserved S2 region, rather than to other S regions, of these CoVs, and all show low or no neutralizing activity [87, 101]. Sequential immunization of llamas with SARS-CoV-2, MERS-CoV, and SARS-CoV S proteins, resulted in triple cross-reactive S3_29 and 6A1, which recognize epitopes at the top and stem-helix regions of the S2 domain, respectively [101]. These nanobodies simultaneously reacted with the S proteins of SARS-CoV-2, SARS-CoV, and MERS-CoV, but without neutralizing these viruses [101]. A phage display library from sharks immunized with SARS-CoV-2 S2 protein was used to derive S2G8, S3A10, and S4A9, which cross-react with SARS-CoV-2, SARS-CoV, and MERS-CoV S2 regions, and all demonstrate low neutralizing activity against a pseudotyped SARS-CoV-2 original strain [87].

The structures of some cross-reactive or cross-neutralizing nanobodies, such as aSA3, have been solved (Fig. 6C) [109]. These structures reveal critical amino acid residues within the S protein or RBD fragment that are recognized by these nanobodies, providing important information for designing universal therapeutic agents against pathogenic CoV infections.

### Humanizing coronavirus-targeting nanobodies

Several CoV-targeting nanobodies have been humanized to reduce potential immunogenicity in humans without decreasing their binding affinity and/or neutralizing activity. VHH72 was humanized by introducing the S56A mutation and by replacing the N-terminal glutamine with a glutamic acid codon [105]. The humanized nanobody humVHH_S56A shows increased binding affinity for the SARS-CoV-2 RBD, preventing its binding to the ACE2 receptor, and improving its ability to neutralize pseudotyped SARS-CoV-2 and SARS-CoV [105]. The Fc-fusion format (harboring LALA mutations) of this nanobody prophylactically or therapeutically prevents SARS-CoV-2 infection of challenged hamsters, with either undetectable or very low viral titers and viral RNA levels in the lungs, nose, and/or throat [105].

### Coronavirus-specific human single-domain antibodies

Human single-domain antibodies specific to the RBD fragment or no-RBD S protein of SARS-CoV-2 have been developed, being able to maintain similar advantages of nanobodies (such as small size and nanomolar binding affinities), as well as neutralizing activity and/or protective efficacy against infection of SARS-CoV-2 original strain and variants [120–123]. Several of these antibodies bind to both the RBD and S1 proteins of SARS-CoV-2, recognizing different epitopes on the RBD, whereas n3072 binds to the non-RBD S1 region [123]. A bispecific antibody bn03, which consists of two human single-domain antibodies recognizing different epitopes on the RBD of Omicron variant, presented higher and broadly neutralizing activity than their respective cocktails, and protected hACE2 transgenic mice against SARS-CoV-2 infection via inhalation delivery [120].

### Half-life of coronavirus-targeting nanobodies

Monomeric nanobodies targeting CoVs have a low molecular weight and are cleared quickly from bloodstream; therefore, they need to be fused to other tags to extend their serum half-life. The Fc region of human IgG is the most common tag used to achieve this. Fc-fused Nanosota-1C maintained strong affinity for the RBD after 10 days of intravenous injection, whereas its counterpart lacking Fc was detectable for only several hours [73]. Similarly, different from the non-Fc format of NbMS10, the MERS-CoV-2 RBD-targeting NbMS10-Fc nanobody still maintains strong binding affinity 10 days after intravenous injection, and unlike non-Fc fused VHH-83, Fc-fused HCAb-83 maintains a serum half-life of about 4.5 days after intraperitoneal inoculation [116, 118]. In addition to a human Fc tag, human serum albumin (or nanobodies targeting this protein) can also be fused to SARS-CoV-2 RBD-targeting nanobodies to increase the nanobody’s half-life, although this appears to be dependent on the route of administration [124]. Nb_15_-Nb_H_-Nb_15_, which was constructed by fusing two SARS-CoV-2 RBD-targeting Nb15 nanobody molecules with a nanobody targeting human serum albumin, was stable for 7 days in the lung of mice after administration via the intranasal route, whereas it was cleared quickly when administered via the intraperitoneal route [124].

## Challenges and strategies for improving coronavirus-targeting nanobodies

The RBD of CoVs contain highly potent neutralizing epitopes and are thus the major target for development of nanobodies. Indeed, as described in the above sections, nanobodies targeting the RBD of three highly pathogenic CoVs, particularly SARS-CoV-2, have been developed, and show higher neutralizing activity than those targeting other regions such as the NTD and S2 of S protein. The SARS-CoV-2 RBD has undergone continuous mutation. This means that nanobodies targeting the original strain or early variants may show significantly reduced, or complete loss of, neutralizing activity against recent variant strains; some key mutant residues have played a critical role in this process. Thus, it is necessary to constantly design new nanobodies with high neutralizing activity and efficacy against these variants. Notably, structure-guided design or molecular modeling-based approaches, through engineering of current RBD-targeting nanobodies with known structures or recognizing epitopes, facilitate rapid development of novel and potent nanobodies against current and future dominant SARS-CoV-2 variants, which will save time and reduce costs of the re-immunization process [94, 105].

Compared with the RBD, the S2 region of the CoV S protein shows much higher sequence homology between strains/variants, making it an important target for development of universal SARS-CoV-2 or pan-CoV therapeutic nanobodies with broad-spectrum activity. Despite of the fact that several such nanobodies targeting SARS-CoV-2, SARS-CoV, and MERS-CoV S2 subunits have been identified, they show low or no neutralizing activity against these viruses, thus new nanobodies with broadly and potent efficiency need to be developed to prevent the emergence of new SARS-CoV-2 variants and CoVs with pandemic potential. This may be achieved, at least partially, by identifying new and highly conserved neutralizing epitopes within the S2 and other regions of the S protein, and by guiding rational design using structure-based strategies.

Bivalent, trivalent, and even multivalent nanobodies targeting the above-mentioned CoV S proteins show increased neutralizing potency and/or protective efficacy against viral infection. It is advisable therefore to construct such nanobodies by linking nanobodies that target different epitopes within the RBD and/or other regions of the S protein to increase efficiency against SARS-CoV-2 variants and pandemic CoVs. A human Fc tag is commonly added as a fusion partner to improve binding affinity, neutralizing activity, protection against virus challenge, and half-life. The Fc-fusion portion can be mutated by introducing LALA substitutions (L234A/L235A) to reduce potential side effects caused by the effector functions, or engineered to include YTE (M252Y/S254T/T256E), LS (M428L/N434S), or other mutations (Q311R/M428L) to further extend serum half-life and increase bioavailability of modified nanobodies [105, 125–128].

Apart from constructing multivalent nanobodies, combinatorial nanobody therapies could be utilized as an additional strategy to improve nanobody’s prophylactic and therapeutic efficacy. Nanobodies with synergistic effects should be selected for such treatments, and pulmonary or oral inhalation administration is recommended if nanobodies are to be delivered to the lungs; this will further improve their protective efficacy against SARS-CoV-2 and other CoV-mediated lung diseases. Alternatively, nanobodies with potent efficacy can be co-administered with other therapeutic agents with different mechanisms of actions (such as neutralizing monoclonal antibodies and antiviral drugs) to prevent and treat CoV infection.

## Conclusions

The three highly pathogenic CoV described herein have had significant negative effects on global public health and continue to post a threat. Among all viral proteins, the S protein, particularly its RBD region, is an important therapeutic target. Nanobodies are effective therapeutic agents that prevent and treat CoV infection. This review describes the viral proteins including the S protein, and systemically summarizes currently developed nanobodies that target these pathogenic human CoVs. We briefly describe potential strategies to improve their potency against SARS-CoV-2 variants and other CoVs with pandemic potential. Although a variety of CoV-targeting nanobodies have been developed, a few have entered human clinical trials, and none are approved for use in humans. More funding input is anticipated to progress nanobodies with high potency and broad-spectrum efficacy against multiple SARS-CoV-2 variants or all three highly pathogenic CoVs into clinical studies. Once confirmed to be effective, these nanobody candidates, or the cocktails of different candidates, can be used for immediate application or future treatment of emergent variants and pandemic CoV infection. Overall, the approaches described in this review will provide useful guidance for designing effective nanobody-based therapeutics against CoVs in particular, and other pathogenic viruses in general.

## Data Availability

No datasets were generated or analysed during the current study.

## Abbreviations

ACE2: angiotensin converting enzyme 2
CoV: Coronavirus
COVID-19: CoV Disease 2019
CDR3: complementarity determining region 3
DPP4: dipeptidyl peptidase 4
E: envelope
Fab: fragment antigen-binding region
FP: fusion peptide
HCAbs: camelid heavy-chain antibodies
HR1 and HR2: heptad repeat 1 and 2
M: membrane
MERS-CoV: Middle East respiratory symptom-CoV
N: nucleocapsid
Nb: nanobody
NTD: N-terminal domain
NSPs: non-structural proteins
RBD: receptor-binding domain
S: spike
SARS-CoV-2: severe acute respiratory symptom CoV-2
scFv: single-chain variable fragment
6HB: six-helix bundle
VH: variable heavy chain
